# Rating neighborhoods for older adult health: results from the African American Health study

**DOI:** 10.1186/1471-2458-8-35

**Published:** 2008-01-25

**Authors:** Elena M Andresen, Theodore K Malmstrom, Fredric D Wolinsky, Mario Schootman, J Philip Miller, Douglas K Miller

**Affiliations:** 1 Department of Epidemiology and Biostatistics, College of Public Health and Health Professions, University of Florida Health Sciences Center, PO Box 100231 Gainesville, FL 32610, USA; 2 Department of Neurology & Psychiatry, School of Medicine, Saint Louis University, 1438 South Grand Boulevard, St. Louis, MO 63104, USA; 3 Iowa City Veterans Affairs Medical Center, Highway 6 West, Iowa City, Iowa 52246, USA; 4 Department of Health Management and Policy, College of Public Health, University of Iowa, 200 Hawkins Drive, E205 General Hospital, Iowa City, Iowa 52242, USA; 5 Departments of Medicine and Pediatrics, School of Medicine, Washington University, Campus Box 8504, 660 South Euclid Avenue, St. Louis, MO 63110, USA; 6 Division of Biostatistics, School of Medicine, Washington University, Campus Box 8067, 660 South Euclid Avenue, St. Louis, MO 63110, USA; 7 Indiana University Center for Aging Research, School of Medicine, Indiana University, IN, USA; 8 Regenstrief Institute, Inc., 410 West 10th Street, Suite 2000, Indianapolis, IN 46202, USA

## Abstract

**Background:**

Social theories suggest that neighborhood quality affects health. Observer ratings of neighborhoods should be subjected to psychometric tests.

**Methods:**

African American Health (AAH) study subjects were selected from two diverse St. Louis metropolitan catchment areas. Interviewers rated streets and block faces for 816 households. Items and a summary scale were compared across catchment areas and to the resident respondents' global neighborhood assessments.

**Results:**

Individual items and the scale were strongly associated with both the catchment area and respondent assessments. Ratings based on both block faces did not improve those based on a single block face. Substantial interviewer effects were observed despite strong discriminant and concurrent validity.

**Conclusion:**

Observer ratings show promise in understanding the effect of neighborhood on health outcomes. The AAH Neighborhood Assessment Scale and other rating systems should be tested further in diverse settings.

## Background

Researchers and practitioners have long noted the correlation between the social disadvantage of populations and individuals, and their health. In the U.S.A., social disadvantage is usually operationalized as individual socioeconomic status (SES) and measured by such individual factors as income, poverty, education, and other social circumstances. Nonetheless, a number of aspects of the built and natural environments of the places people live have come under increasing scrutiny. Researchers and practitioners embrace the concept that "place matters" in producing disparate health outcomes [1-7]. Despite the theoretical importance of the effects of neighborhoods on health outcomes, few studies incorporate independent observer ratings of neighborhood conditions. Methods and measures that objectively rate people's physical neighborhoods have not been accompanied by published documentation of development or by methodological tests to gauge possible problems in validity and reliability.

Disparate theories and applied research examples do not produce consensus on which contextual factors to measure, nor agree on a uniform size and definition of the appropriate spatial size that influences health [2,3,8-14]. In our ongoing cohort study of African American adults in the St. Louis metropolitan area, we theorize that, in addition to individual SES characteristics, place matters [5]. The overall study goals address issues of health disparities by investigating risks for adverse outcomes within an African American cohort, rather than focusing on comparisons with other race and ethnic groups. This approach eliminates the issue of confounding by race for our cohort results. We explicitly incorporated multiple spatial levels in sampling and in our measures. In addition to sampling from two geographic areas of different composite SES (below), we included direct observer ratings of neighborhoods. For the purposes of our study, the block on which the respondent lived was used as a proxy for a larger implicit level of "neighborhood."

Neighborhood assessment at the cohort inception (wave one) was based on an evaluation of the external appearance of the block on which the respondent lived. Survey team members completed this assessment during the process of household enumeration in March and April of 2000. The 5-item scale was based on work by Krause [15] and had been used specifically in research with older adults. The component items included the condition of houses, amount of noise (from traffic, industry, etc.), air quality, condition of the streets, and condition of the yards and sidewalks in front of homes, and each item was rated excellent (1 point), good (2 points), fair (3 points), or poor (4 points). This assessment tool had acceptable psychometric properties, but we were not satisfied with its discriminative ability or its inter-rater reliability [16]. Further, we found a marked tendency for raters to choose "good" ratings for most items (48% to 68% of all ratings among the five items were rated "good"). This also impacted the summary scores: 38% of all scale scores were 10.0 points (all 5 items rated "good"). Finally, when modeling the association of neighborhood on scale scores, we found that interviewers and characteristics of interviewers also contributed to different ratings. As part of the fourth wave, which was an in-home assessment (2004), we strove to select and test an improved observer rating method for future analysis of the impact on health outcomes. We performed a small pilot to select a candidate method, and then conducted three phases of analyses to test the instrument. Phase 1 assessed items and produced a potential brief scale; phase 2 tested the discriminant and concurrent validity of items and the scale; and phase 3 examined potential interviewer effects.

## Methods

### Participants

The baseline sampling strategy for the African American Health (AAH) study involved two St. Louis geographic areas that differ widely in SES [17-23]. One catchment area is a poor, predominantly African American inner city neighborhood where 24% of AAH respondents report annual incomes under $10,000. The second catchment area is a suburban, integrated neighborhood, where 8% of AAH respondents report annual incomes under $10,000. Households were sampled based on an enumeration of all housing units in the two catchment areas with at least 10% African American households by the 1990 Census. Characteristics of this cohort have been described in detail elsewhere [19]. Briefly, a total of 998 African American adults aged 49 to 65 (76 percent of eligible subjects) were recruited from random sampling of households from two strata: a poor, inner-city area of St. Louis (n = 463), and the near Northwest suburbs (n = 535). The cohort included 41.8% men at baseline and had a mean age of 56.8 years.

Most participants resided on a block on which no other AAH participant resided (65.9%). Only 3.6 percent of block faces contained five or more participants. For these analyses, we did not analyze this nesting between and within block faces because there was not enough clustering of participants within block faces to support a robust multi-level analytic approach [17]. In addition, 73.1 percent of AAH respondents lived at the same address for more than five years before their baseline interview, and nearly three-quarter (74.6%) lived at the same address during all three years of follow-up [17].

In-person baseline interviews were conducted in 2000–2001, annual telephone interviews were conducted during waves two (2002) and three (2003), and for wave four (2004), in-person interviews were conducted with 90.0% of the surviving members of the cohort [17]. In preparation for the in-home assessment of cohort members, we asked experienced interviewers on our team for their feedback about the rating tool we used in wave one [15,16]. They indicated that the scale lacked specific and objective criteria to assess and chose rating levels. They also recommended that more thorough training, use of visual examples, and question-by-question guidelines would be required before consistent ratings among interviewers would be possible. In addition to the five-item assessment instrument from wave one, we pilot tested two other instruments with three core experienced AAH interviewers during pretesting of the in-home assessments. We used an 11-item form rating inside subjects' homes and their neighborhood that was adapted from the National Longitudinal Survey of Youth [24]. We also piloted a 20-item block assessment adapted and simplified from the Project on Human Development in Chicago Neighborhoods [10]. We conducted preliminary training on all three forms using pictorial examples of items (e.g., street conditions, litter). Thirty-one subject neighborhood ratings using all three forms were piloted during several weeks, including written qualitative feedback by the interviewers. These experiences were then debriefed with members of the investigator team and field supervisor. Interviewers universally considered the adapted "Chicago" form to be the easiest to rate and the most objective of the three. We therefore adopted this form.

### Instrument

The adapted Chicago form obtains information about the neighborhood generally (e.g., noise, dust, street) as well as information about the two block faces of the street of each research respondent (e.g., housing conditions, presence of security measures, presence of commercial property). We trained interviewers using digital photo images of neighborhood conditions with variations in conditions that spanned the full range of the rating levels. After further development and pretesting of the training protocol, including feedback from the interviewer team leaders and field supervisor, we created a detailed training manual and supportive material for the final wave four protocol. Twenty-six interviewers were trained on specific aspects of the rating protocol during approximately 2.5 hours of a weeklong research protocol-training program. A follow-up refresher session was conducted about a month after initial training. Prior to the refresher session, we reviewed the first 269 results of the neighborhood assessments and found satisfactory variation in item responses. We were specifically concerned with the possibility of very frequent ratings for variables that asked about housing and street qualities, and found no strong pattern of common ratings. During the field interview period, team leaders met weekly with their group of interviewers, at which time neighborhood rating was discussed, as needed. Through this mechanism, any uncertainty in the rating methods and unusual circumstances were identified and remedied quickly, and coding clarifications were circulated to all interviewers.

Interviewers usually completed the neighborhood assessment following a scheduled wave four, in-home visit that averaged 99 minutes. If the interviewer expected the post-interview period to fall after dusk, the neighborhood assessment was performed in daylight hours before the appointment. Interviewers were assigned to conduct assessments in both catchment areas (inner city, suburbs). A few assessments (4.3%) were skipped when the respondent was interviewed at a site that was not his/her home, or if the interviewer felt unsafe staying in the neighborhood for the assessment. All study procedures were approved by the supervising academic institutions' human subjects review committees (IRBs).

The original Chicago Neighborhood rating system incorporated assessments of a full four-sided block, and the four streets and both sides of each of the streets of the block (eight block face assessments) [10]. We used a simplified version that incorporated five questions about the street the respondent lived on (traffic volume, street condition, noise, smells, dirt/dust) and 15 questions that were completed for both the block face the respondent lived on (Face A) and the opposite side of that street (Block Face B). Block face questions asked about the presence of general litter and garbage as well as specific items (cigarette products, alcohol containers, abandoned car, condoms, needles). Questions included the presence of graffiti, security measures on residences and commercial property, types of residential and business land use, condition of residential and business property, signs for tobacco and alcohol products, for-sale signs, neighborhood crime programs, vacant lots, parking lots, and the presence of recreational faculties (parks, etc). Interviewers provided ratings on simple Likert-type scales (e.g., for traffic volume, four levels from none to heavy) and simple checks for presence of items (e.g., abandoned car, empty beer or liquor bottles, etc). The full survey and training materials are available from the authors on request.

### Analysis

A total of 84 descriptive variables of each respondent's neighborhood was available, including multi-item checklists and both block faces for 15 items. In phase one, we assessed items for frequencies and response categories, finalized classification rules (e.g., collapsing categories), and conducted exploratory factor analyses and scale internal consistency (coefficient alpha) tests for composing multi-item scales. A series of exploratory factor analyses for block face A items was done to assess the item properties of the neighborhood ratings and develop a neighborhood rating scale. Factor analyses were then repeated using items from block face A + B and compared to the results of the initial factor analyses and internal consistency.

In phase two, we examined the discriminant and concurrent validity of the items and the seven-item summary scale that resulted from phase one. We hypothesized that the scale resulting from phase 1 would exhibit discriminant validity and would produce substantially lower scale scores (better conditions) in the suburban catchment area. For concurrent validity, we compared the scale scores to the resident subjects' rating of their own neighborhood. Each of these analyses was conducted first using data from Block Face A only, and then the combined Block Face A + B ratings. The inner-city catchment area, with poorer levels of SES, was expected to produce higher (worse) total and item scores. Item percentages were compared by chi-square, and means compared by t-tests. We compared item and scale agreements from the two block faces with chi-square for categorical items, and intra-class correlations (ICC) for the summary scale. For convergent validity, interviewer ratings were compared to a global rating of the neighborhoods provided by resident study subjects, who were asked, "All things considered, rate your neighborhood as a place to live. Would you say it is excellent, very good, good, fair, or poor?" Responses were coded from 1 (excellent) to 5 (poor). Neighborhood was self-defined by study subjects. The average score of interviewer raters was compared for linear trend across the categories of subjects' global neighborhood ratings using analysis of variance.

In phase three, we investigated potential interviewer effects on scale scoring. We constructed a linear regression model predicting the total score for the seven-item scale that resulted from phase one, first using Block Face A only, and then the composite of Block Faces A + B. We grouped interviewers into two categories of experience. Nine interviewers had been involved in the study assessments or training activities for this study during some or all of the prior three years, and nine interviewers were new to the study and new to research interviewing. We used forced variable entry with catchment area, interviewer experience, and individual interviewer as dummy variables comparing to a referent interviewer, selected as the person with the maximum completed assessments (n = 102). We excluded an additional set of new interviewers (7/25) from this analysis who had not completed at least five assessments in each catchment area. Analyses were conducted using SPSS Version 12.0 [25].

## Results

### Phase 1: Neighborhood rating scale in AAH

Neighborhood ratings were completed for nearly all subjects who agreed to the in-home interview: 94.6% in the city (n = 364 of 385) and 96.4% in the suburbs (n = 452 of 469). Each assessment took approximately five minutes. Examination of item and response category frequencies led to a number of modifications of the assessment instrument. Fortunately, interviewers found no drug paraphernalia, and only noted one condom among 816 assessments (408 unique blocks with two block faces rated). There were too few recreational faculties to include a rating of their condition, and this item was dropped. Only 13 block faces included any type of graffiti, and gang and other graffiti categories were collapsed to "any" and "no" graffiti. In addition, some extreme ratings were rare, and response categories were collapsed. For example, noxious smells and dirt/dust categories were collapsed to "none" and "any" categories. Categories of how many residences and buildings had security measures were also were collapsed to "none" and "any" categories. Land use details were simplified to residential compared to multi-use neighborhood blocks. Residential housing types were classified as detached single family, multi-family (duplex, condo, row house), private apartment buildings, and public housing buildings. Table 1 displays the items retained, and coding levels for each. In all analyses reported below, the addition of information from block face B provided no substantive improvement of the results. Therefore, tables provide only results using block face A.

**Table 1:** Items & Scales of the Chicago Neighborhood Rating Method from Two Areas of Metropolitan St. Louis.

	**General conditions**		
**Item/Scale**	**Overall n = 816**	**Inner City n = 364**	**Suburbs n = 452**

Rating not done %	4.4%	5.5%	3.6%
**Items rating entire street**			
*Traffic volume none to heavy (Mean 0–3 ± SD)	1.04 (± 0.9)	1.32 (± 0.9)	0.82(± 0.8)
*Street condition very good to poor (Mean 0–3 ± SD)	1.16 (± 0.8)	1.39 (± 0.8)	0.98(± 0.8)
*Noise very quiet to very noisy (Mean 0–3 ± SD)	0.71 (± 0.8)	0.89 (± 0.8)	0.56 (± 0.7)
Smells % yes	2.6%	3.3%	2.0%
Dirt & dust % yes	1.1%	1.9%	0.4%
**Items rated on block face of respondent's residence**			
Abandoned car % yes	1.7%	2.7%	0.9%
*Beer/liquor bottles % yes	9.1%	16.2%	3.3%
*Cigarettes % yes	16.2%	24.7%	9.3%
*Garbage, litter none to heavy (mean 0–3, ± SD)	0.53 (± 0.8)	0.86 (± 0.9)	0.26 (± 0.5)
Graffiti % yes	1.0%	2.2%	0.0%
Neighborhood crime signs % yes	4.5%	3.3%	5.5%
Security signs % yes	44.9%	38.5%	50.0%
For sale signs % yes	7.7%	8.2%	7.3%
Commercial property % yes	8.2% (67)	14.6% (53)	3.1% (14)
% Poor/fair condition	2.9%	5.8%	0.7%
% With pull down blinds/iron gates	3.1%	6.3%	0.4%
% With security bars/grates/boards	3.6%	7.1%	0.7%
Primary housing type			
% Single family	65.1%	42.6%	83.2%
% Private multi family	16.9%	34.9%	2.4%
% Private apartments	12.9%	12.9%	12.8%
% Public housing	5.1%	9.6%	1.5%
*Residential condition very good to poor (Mean 0–3 ± SD)	1.11 (± 0.9)	1.55 (± 0.9)	0.75 (± 0.7)
Security bars/grates on residences % yes	27.4%	47.8%	10.9%
Recreational facilities % yes	1.3%	1.4%	1.3%
*Scale: traffic, street condition, noise, beer, cigarettes, garbage, residential unit condition (Mean 0–15 ± SD)	4.81(± 3.2)	6.44 (± 3.0)	3.50 (± 2.7)

* Items sum for the rating scale (range 0–15 points for Block Face A). Higher scores represent worse neighborhood conditions.

SD = standard deviation

Factor analyses of block face A items resulted in a single factor scale including the following 7-items: traffic, street condition, noise, beer/liquor bottles, cigarettes, garbage, and residential unit condition. No other items combined into multi-item scales with sufficient factor loadings, and this summary scale was retained for further analysis. The single forced factor accounted for 43.5% of variance in the items with factor loadings ranging from 0.47 to 0.80. Specific factor loadings were traffic (0.47), street condition (0.65), noise (0.53), beer/liquor bottles (0.65), cigarettes (0.70), garbage (0.80), and residential unit condition (0.76). The alpha for the 7-item block face A scale was 0.75. Deleting the item with the lowest factor loading (traffic) reduced the alpha very slightly to 0.74. A subsequent factor analysis of the same scale using the block face A + B results specifying a single factor solution yielded similar scale properties, accounting for 44.9% of variance in the items with factor loadings ranging from 0.49 to 0.80 and an alpha of 0.73. The means and standard deviations for the scale using block face A are provided in Table 1. The seven-item scale is shown in the Appendix.

### Phase 2: Discriminant and concurrent validity

The scale and most items showed strong and consistent differences in rating between the inner city and suburb neighborhood ratings whether using data from Block Face A only, or Block Face A + B combined. For example, using Block Face A data, the scale scores were nearly 3 points higher (worse) for inner city compared to suburban neighborhood street (scale means 6.44 vs. 3.50, respectively). Among items, there were striking differences in the presence of problems like beer/liquor bottles (16.2% inner city versus 3.3% suburbs) and cigarette litter (24.7% versus 9.3%). There were some items where this pattern appeared to be reversed (the observations were more common in the suburbs), but these items were also related to expected catchment area neighborhood composition. For example, while security bars and grates were more common in the inner city, neighborhood crime signs (indicating neighborhood collaborations) and security signs (for formal electronic systems and surveillance contracts) were more common in the suburbs. The condition of both residential and commercial structures was better (lower scores) in suburban ratings. In addition, single-family housing was the predominant type in the suburbs (83.2% for Block Face A) compared to the inner city, where it represented less than half (42.6%) of housing. The only substantive difference in results using Block Face A only or both Block Faces A + B was that, given the possibility of affirmative answer for either block face, percentages were higher for conditions when both block faces were combined.

Table 2 presents the results of testing convergent validity of interviewer rater scale scores and resident subjects rating, and also a more detailed view of city and suburb differences. Comparison of the global rating by AAH subjects to the interviewer rating scale demonstrated a striking trend of increasing scale scores with lower ratings moving from an average of about 3 points for subjects who rated their neighborhood as "excellent" to about 7 points for ratings of "poor" (Table 2). These strong and significant trends were also apparent when separating the results by City and Suburban strata.

**Table 2:** Seven-Item Interviewer Observed AAH Neighborhood Scale* Results Compared to Residents' Global Rating of their Neighborhood.

	**Mean Interviewer Rated Scale Scores**		
**Resident Subjects' Global Rating of their Neighborhood**	**Total Sample**	**Inner City**	**Suburbs**

Excellent	2.96	4.92	2.40
Very Good	3.66	5.38	3.03
Good	5.14	6.51	4.00
Fair	6.18	7.02	4.71
Poor	6.98	7.07	6.60
*p-value for linear trend*	*< .001*	*< .001*	*< .001*

* Sum of the seven-item rating scale (range 0–15 points for Block Face A). Higher scores represent worse neighborhood conditions.

### Phase 3: Interviewer effects

Eighteen (72%) of the 25 interviewers completed 5 or more assessments in each catchment area. The seven interviewers with fewer than five assessments/area completed 15.8% (135/816) of the neighborhood assessments. There was no association of interviewer experience and scale score (β coefficient 0.326, p = 0.135), and this variable was dropped from the final model. Table 3 displays the results of the analysis of interviewer effects using Block Face A scale ratings only. There were substantial interviewer differences in mean scores, although the strongest relationship with scale score was catchment area (city versus suburbs). Using only Block Face A, eight interviewers varied significantly (p < .05) from the reference interviewer, and using Block Faces A + B, ratings differed significantly for 11 interviewers (data not shown). In an additional analysis we repeated this modeling using Block Face A and including only interviewers (6/25) with at least ten observations in both catchment areas, with minimal changes in the number and magnitude of the interviewer differences. We also introduced the resident subjects' global neighborhood rating variable (analysis of this global rating also shown in Table 2) to potentially account for some real differences in the neighborhoods rated by interviewers, also with minimal changes in the magnitude of score differences among interviewers. Finally, in an ad hoc analysis, we constructed an interaction model of interviewer by catchment area and added this to the regression model in Table 3. Of the 17 interactions, two were statistically significant (p < .05): interviewer 10 provided worse (higher scores) ratings overall, but also significantly worse for inner city ratings; interviewer 13 provided better (lower) ratings overall, but they were significantly worse (higher scores) in the city ratings. The addition of these interaction terms changed the R^2 ^for the model slightly: for the main effect model in Table 3 it was 0.320, and for the model with two interaction terms it was 0.332.

**Table 3:** Interviewer Effects on Neighborhood Scale Scores.

	**Neighborhood Scale**^+^	
**Variable**	B coefficient*	p-value

*Constant*	*3.630*	*< .001*
Catchment area Inner City vs. Suburbs	3.405	< .001
**Interviewers (vs #1)**		
Interviewer 2	1.845	.001
Interviewer 3	-1.554	.003
Interviewer 4	0.607	.158
Interviewer 5	-1.336	.033
Interviewer 6	-1.105	.015
Interviewer 7	-1.007	.065
Interviewer 8	-1.098	.053
Interviewer 9	-0.979	.070
Interviewer 10	2.623	< .001
Interviewer 11	-0.715	.368
Interviewer 12	-0.911	.079
Interviewer 13	-0.473	.313
Interviewer 14	-1.173	.009
Interviewer 15	-1.927	< .001
Interviewer 16	-0.299	.707
Interviewer 17	-0.077	.898
Interviewer 18	-1.742	.002

^+ ^Scale includes summary of items measuring traffic, street condition, noise, beer, cigarettes, garbage, and residential unit condition. Scale ranges from 0–15 points. Higher scores represent worse neighborhood conditions.

* Unstandardized beta coefficients

R^2 ^for this model = 0.320

## Discussion

This study demonstrates that observer ratings of neighborhood characteristics achieve substantial discriminant and convergent validity. This was evident in both individual items, like housing stock, and the seven-item scale we constructed in phase one. The addition of observer ratings of a second block face did not provide any substantive improvement over the information provided by a single block face, suggesting that the time and labor of our neighborhood assessment tool can be reduced. The seven-item scale produced a striking difference of 2.94 points between inner city and suburban neighborhood rating within a scale range of 0–15 points. This approximately 3-point difference is equivalent to one item with multiple response levels (e.g., noise, residence condition) changing 3 points, or the two dichotomous items (presence of liquor, cigarettes) changing from "no" to "yes" and one other item changing one category, etc. This reflects a real difference between the neighborhoods, and not an artifact of measurement. In addition, the ratings by interviewers showed a strong linear relationship to subjects' own global ratings of their neighborhood in both catchment areas.

Neighborhood and SES are part of the conceptual framework in the AAH cohort. Consequently, recruitment was targeted to maximize neighborhood diversity, and neighborhood effects are not confounded by race because the cohort is composed entirely of African Americans. Because this is a study of one minority group of mature adults in a single metropolitan area, the results may not extend to other urban areas and populations. In particular, it is possible that confounding by race might be present in a multi-race study due to racial differences in how residents rate their own neighborhoods. Finally, the spatial size that we used in this study may not be appropriate for other studies with different populations or objectives.

Despite substantial training, ongoing monitoring, and the generally positive psychometric results of the neighborhood assessments by items or the scale, we did not eliminate individual interviewer rating variability. Our adapted scale and items demonstrated more item response variation than our prior work with a simple five-item scale [16], and we detected no problem with a response "set" as in the prior rating scale where we found that a large percentage of neighborhood ratings were placed at the same level of "good" conditions. In addition, interviewers reported that the new rating scale was relatively easy to use because of clear criteria for classification of what they observed and because the items were relatively objective (i.e., presence of alcohol containers, security bars, traffic volume, etc).

The persistence of an interviewer effect for rating neighborhoods is troubling, and not easy to explain. It does not appear to be dependent on which neighborhood was rated, and our test of interaction between interviewer and area yielded only two possible interactions – a finding that should be viewed with caution due to the multiple testing and the lack of a consistent pattern. Gauvin and colleagues [26] reported a small amount of variation (4% to 14.8%) from observers based on four pairs of trained observers of randomly selected Montreal street segments. Their rating system was quite different from the one we report here, and the largest variability was for the dimension of "activity friendliness." In our own test of the five-item neighborhood rating measure we used during the baseline of the AAH cohort, we found adequate inter-rater reliability despite interviewer effects [16]. In another St. Louis area study that audited street segments for community indicators to improve physical activity, inter-rater reliability results were variable among measures of environmental attributes ranging from built environment items to social and aesthetic items [27]. We are unaware of any other published results of observer neighborhood rating scales that can provide evidence that this problem of interviewer effects is relatively common, or if it is a result of our choice of AAH catchment areas, our training, or the instruments. Neighborhood rating systems, including the AAH Neighborhood Assessment, need to be tested in diverse settings. In addition, additional formal testing of interrater reliability is necessary to assess the magnitude of inconsistency among raters.

## Conclusion

Overall, the AAH Neighborhood Assessment and its resulting seven-time scale produced strong differences in neighborhood scores representing real differences between areas with known SES differences. Observer ratings of neighborhoods show promise as a measure of neighborhood and the effect of neighborhood conditions on health outcomes.

## Competing interests

The author(s) declare that they have no competing interests.

## Authors' contributions

All authors contributed to the concept, design, and/or analysis and interpretation of data. The corresponding author completed the initial draft and all authors assisted with revising the manuscript. All authors have reviewed the final version of the manuscript and approved it for publication.

## Appendix

### African American Health Seven-Item Neighborhood Assessment scale

*The first 3 questions refer to the full block and street on which the respondent lives*.

1. Volume of traffic

0. No Traffic

1. Light (occasional cars)

2. Moderate

3. Heavy (steady stream of cars)

2. Condition of the street

0. Under construction

1. Very poor (many sizeable cracks, potholes, or broken curbs)

2. Fair

3. Moderately good (no sizeable cracks, potholes, or broken curbs)

4. Very good

3. How noisy is the street?

0. Very quiet – easy to hear almost anything

1. Fairly quiet – can hear people walking by talking, though you may not understand them

2. Somewhat noisy – voices are not audible unless very near

3. Very noisy – difficult to hear a person talking near to you

*Items 4 through 7 are answered based on observations of the side of the street on the block where the respondent lives (block face)*.

4. Are empty beer or liquor bottles in street, yard, or alley present?

1. Yes

0. No

5. Are there cigarette or cigar butts or discarded cigarette packages on the sidewalk or in the gutters?

1. Yes

0. No

6. Is there garbage, litter, or broken glass in the street or on the sidewalks?

0. None

1. Light (some visible)

2. Moderate

3. Heavy (visible along most or all of street)

7. In general, how would you rate the condition of most of the residential units in the block face?

0. Very well kept/good condition – attractive for its type

1. Moderately well kept condition

2. Fair condition (peeling paint, needs repair)

3. Poor/Badly deteriorated condition

## Pre-publication history

The pre-publication history for this paper can be accessed here:


